# Disseminated peritoneal cystic Echinococcosis in a patient with HIV: case report

**DOI:** 10.17843/rpmesp.2023.402.12479

**Published:** 2023-06-20

**Authors:** Pedro J. Ruíz-Pérez, Melissa Janet Huayapa-Avendaño, Karla Beatriz Gómez Leyva, Marco A. Rivera-Jacinto

**Affiliations:** 1 Ate Vitarte Emergency Hospital, Lima, Peru. Ate Vitarte Emergency Hospital Lima Peru; 2 Vitarte Hospital, Lima, Peru. Vitarte Hospital Lima Peru; 3 Microbiology laboratory, Universidad Nacional de Cajamarca, Cajamarca, Peru. Universidad Nacional de Cajamarca Microbiology laboratory Universidad Nacional de Cajamarca Cajamarca Peru

**Keywords:** Echinococcosis, Zoonoses, Peritoneal Cavity, HIV, Ultrasonography, Peru

## Abstract

Cystic echinococcosis is a zoonotic infection caused by the larva of *Echinococcus granulosus*, which is capable of invading several organs starting from the human intestine. There are several complications in cases of co-infection with the human immunodeficiency virus (HIV), which are conditioned by the immunosuppressive disease and have poor prognosis. This report aims to describe a case of multi-cystic peritoneal echinococcosis in a patient under antiviral treatment for HIV for almost ten years, who received albendazole, underwent surgery and progressed favorably. This would be the first Peruvian report of a person with HIV and cystic echinococcosis.

## INTRODUCTION

Cystic echinococcosis (CE) is a zoonosis of worldwide impact caused by infection with the larval stage of the tapeworm *Echinococcus granulosus*, found in dogs (definitive host), whose feces may contaminate soil, water and food ^1^^,^^2^. The intermediate host (livestock) and humans become infected from contaminated elements. The larva may infect several organs, mostly liver and lungs, after reaching the intestine and crossing the intestinal wall ^3^.

This disease can also be found in areas where sheep and cattle are raised, and it has high incidence rates in the Mediterranean region, North Africa, southern and eastern Europe, South America, Central Asia, Siberia and western China ^4^. In endemic regions, incidence rates can reach 50 cases per 100,000 inhabitants per year, and its prevalence in specific areas of Peru and other South American countries can vary between 5 and 10% ^5^. In Peru, most cases occur in adult women, approximately 60% have been reported in the departments of Lima, Huancavelica, Junín, Cerro de Pasco and Cusco; most occur in adult women, and one third are cases of pulmonary echinococcosis ^6^.

Coinfection with parasites is usually a severe complication for patients with human immunodeficiency virus (HIV); however, there are few reports of coinfection with CE worldwide ^7^. Therefore, we describe the first case in Peru, highlighting the interdisciplinary management of a disease with rapid progression and multiple complications in immunosuppressed patients.

## CASE REPORT

**Figure 1:**
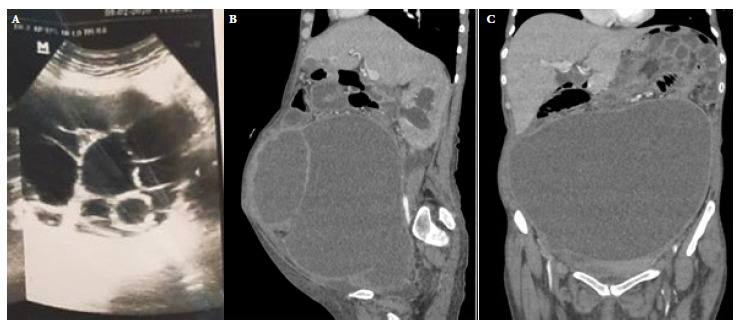
Imaging studies: (A) Ultrasonography performed in 2022: heterogeneous mass, 9 × 10 cm^2^, with multiple cystic images inside, type III according to the Gharbi classification. (B) Sagittal section of multi-slice spiral tomography with contrast. (C) Coronal section of multi-slice spiral tomography with contrast performed in 2022: complex cystic mass of mixed nature, 25 × 19 × 26 cm^3^, with displacement of adjacent intra-abdominal structures and hydroureteronephrosis, type IV according to the Gharbi classification.

A 34-year-old woman from the district of Paucartambo, province of Pasco, Pasco region in Peru, with a history of HIV infection on irregular treatment with dolutegravir/lamivudine/tenofovir since 2013, who reported that one of her children underwent surgery in 2022 due to an abscessed hepatic-splenic hydatid cyst. The patient reported that she currently lives in Lima, but that she had had contact with cattle and dogs in her community of origin. The patient presented mild abdominal pain since 2013, ultrasonography results showed an abdominopelvic multicystic mass of approximately 10 cm at the time. Abdominal pain persisted, and nausea, vomiting, constipation as well as the abdominal diameter increased since 2020. On late 2021, the patient had difficulty with oral tolerance and presented lower limb edema; the Western Blot test showed *Echinococcus granulosus*, so the patient started receiving albendazole 400 mg every 12 hours for 28 days. Figure 1 shows the progressive evolution of the size and complexity of the cystic masses by imaging studies.

**Figure 2:**
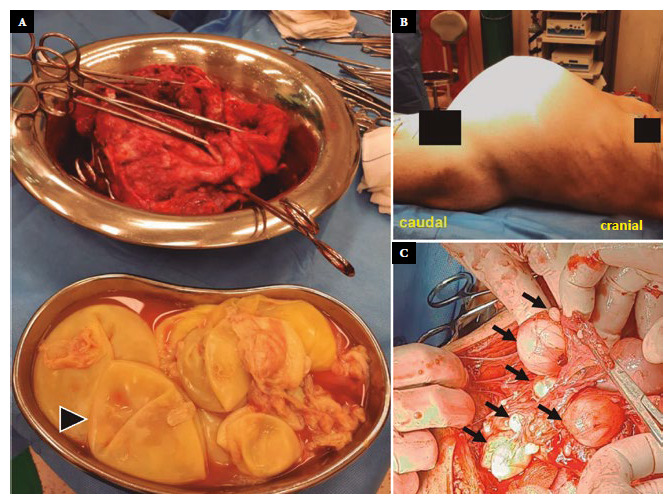
Complex cystic mass. (A) Surgical specimen and vesicular membranes (arrowhead). (B) Abdominal distention caused by intra-abdominal cystic mass. (C) Multiple cystic lesions (arrows) occupying the intestinal mesentery.

On early 2022, the patient was evaluated and admitted to the surgery department, then she received a second course of albendazole. Additionally, before surgery, she received high-protein, hypercaloric supplementation plus immunonutrients and polymaltose iron for four weeks, due to malnutrition and anemia. Subsequently, the patient underwent an emergency exploratory laparotomy due to intestinal obstruction, and complete surgical resection of a complex multicystic mass measuring 35 × 30 × 30 cm^3^, with thickened walls and fused with the greater omentum, extending into the pelvis and retroperitoneum, firmly adhered to the anterior and posterior peritoneum, posteroinferior aspect of the bladder and upper part of the uterus (Figure 2). Multiple cystic lesions smaller than 8 cm were also resected, which were located throughout the mesenteric root of the jejunum, ileum and colon; two silicon drainage tubes were placed in the lateral, intra-abdominal recesses.

The patient remained at the hospital for seven days, during which she received antibiotic therapy (ceftriaxone + metronidazole), a third course with albendazole, transfusion of five red blood cell units due to acute post-surgery anemia, and caloric-protein supplementation, progressing favorably with adequate oral tolerance. The drainage tubes contained serosanguineous fluid, which decreased until they were removed during the follow-up appointment, after hospital discharge.

**Figure 3:**
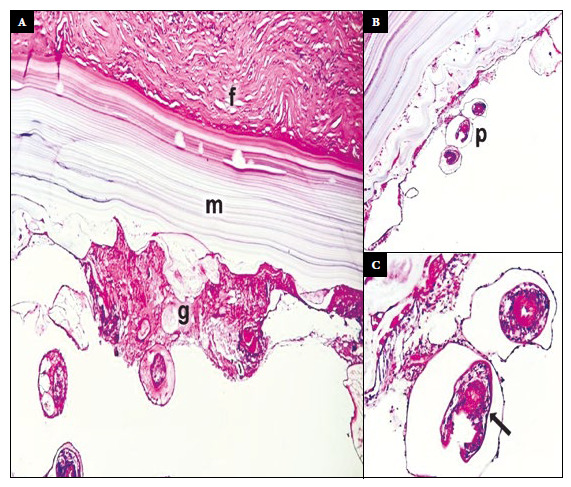
Histopathology of the surgical specimen. Histological sections of the cyst, hematoxylin-eosin staining. (A) The cyst wall had three structural components: fibrous capsule (f), acellular laminated membrane (m) and germinative membrane (g) (10X). (B) Protoscolices (p) sprouting from the germinative membrane (4X). (C) Protoscolices with suckers and hooks (arrow) (40X).

**Figure 4:**
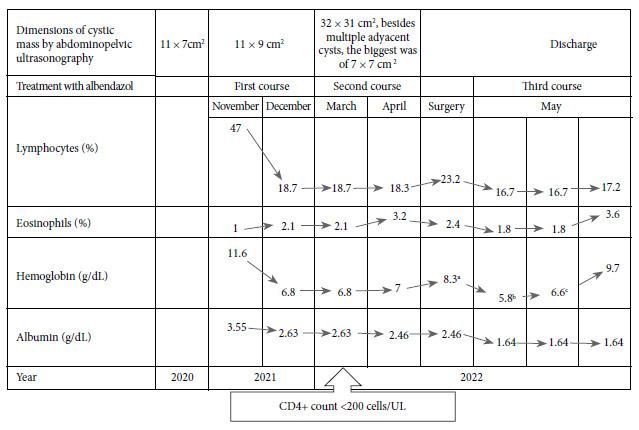
Comparison of pre- and post-surgery laboratory results.

Histopathology showed a complex and viable hydatid cyst (figure 3). The progression of the disease and laboratory results are presented in figure 4. Six months after surgery, the patient carried out her normal activities, adhered to the antiretroviral treatment and attended her periodic follow-up appointments.

## DISCUSSION

CE is endemic in many regions worldwide. It is related to livestock activities which, together with the limited knowledge regarding its transmission mechanism and deficient prevention measures, lead to the persistence of this zoonosis ^4^. The asymptomatic and slow progression of the disease can trigger the abrupt growth of cystic lesions and the involvement of multiple organs in 20% of the cases ^3^.

The most frequent symptom is abdominal pain ^8^, which is almost always associated with complications due to the size of the cyst. In our case, the multicystic mass compressed multiple organs (stomach, intestines, ureters and bladder) due to its size; it also led to the appearance of symptoms such as anorexia, nausea and vomiting, similar to previous reports ^8^^-^^12^. The patient presented stress incontinence and lower limb edema, which are considered to be rare symptoms.

Recently, HIV has been reported to be the most frequent immunodeficient infection in patients with CE ^2^. In addition, some reports show that extrahepatic cystic disease is more frequent in people with HIV ^7^, as has been described in cases with unusual locations such as the brain ^13^, spinal cord ^14^, eyeball ^15^, kidney ^16^, kidney-liver ^17^^)^ and peritoneal ^18^. Most of these cases occurred in women ^2^.

The immunity mechanisms involved in the defense against echinococcosis are characterized by the activation of Th2 cells and a Th1 subset, which leads to the expression of immunoglobulin isotypes (IgG4 and IgE) and eosinophilia ^19^. In our case, transient eosinophilia was found after surgery, which could have been due to the rupture of some cysts during surgical removal ^12^, considering that the hystopathological study reported the presence of viable cysts.

Several studies have reported improvement of symptoms, a decrease in cyst size by imaging tests, and even seronegativity after treatment with albendazole ^1^, this could be due to the effect of antiparasitic treatment in the increase of the production of interferon gamma (IFN-γ) and tumor necrosis factor alpha (TNF-α) by peripheral mononuclear cells ^19^. On the contrary, our case showed an abrupt increase in the size of the cyst with increasingly worsening disease progression, due to gastric, intestinal and ureteral compression, concordant with a decrease in CD4+ T cells (55 cells/uL) during treatment with albendazole. These findings can be explained by the suppression of these cells by the parasitic infection ^20^, which predisposes the uncontrolled proliferation of proto-scolices, accelerated growth and the formation of multiple cysts in HIV-positive patients with altered immunity ^12^, and in this case, in a stage of acquired immunodeficiency syndrome (AIDS).

A systematic review reported that surgery is the main treatment for CE in immunocompromised patients, being performed in more than 70% of cases ^2^. In our case, we opted for surgery along with antiparasitic treatment, because evidence indicates that the combination of both interventions can significantly increase CD4+ T-cell count ^8^^,^^20^, decreasing the associated morbidity. However, mortality is mainly associated with complications of the interventions when lesions reach a considerable size ^3^, in our case, acute anemia due to bleeding during surgery was controlled with transfusions.

The main limitation of this case report was that the CD4+ T-cell count was not updated due to the fact that this test is performed at the National Health Institute, through referral and prior evaluation at the infectious disease department of the Hospital Hipólito Unanue, which takes too much time. In addition, due to the complexity of the lesion, the patient was admitted to different specialties and health institutions, which delayed the surgical intervention. It is possible that both factors could have allowed a better management of the patient.

The patient did not report any recurrence after eight months of follow-up. Therefore, we conclude that combined therapy (surgery + antiparasitic treatment) could be the treatment of choice for the treatment of CE in patients coinfected with HIV/AIDS, with favorable long-term results.
