# Supplementary material for: Disseminated peritoneal cystic Echinococcosis in a patient with HIV: case report
Source: Rev Peru Med Exp Salud Publica. 2023 Jun 20;40(2):236–41. doi: 10.17843/rpmesp.2023.402.12479 (PMC10953647; doi:10.17843/rpmesp.2023.402.12479)
Supplement: Translation

# Translation: Equinococosis quística peritoneal diseminada en paciente con infección Por VIH: reporte de caso

**Authors:** Pedro J. Ruíz-Pérez, Melissa Janet Huayapa-Avendaño, Karla Beatriz Gómez Leyva, Marco A. Rivera-Jacinto

**Affiliations:** 1 Hospital Emergencia Ate Vitarte, Lima, Perú.; 2 Hospital Vitarte, Lima, Perú.; 3 Laboratorio de Microbiología, Universidad Nacional de Cajamarca, Cajamarca, Perú.

**Keywords:** Equinococosis, Zoonosis, Cavidad Peritoneal, VIH, Ultrasonografía, Perú

## Abstract

La equinococosis quística es una infección zoonótica producida por la larva de *Echinococcus granulosus* que es capaz de invadir diversos órganos desde su ubicación en el intestino humano. En los casos de coinfección con el virus de la inmunodeficiencia humana (VIH), existe una diversidad de complicaciones condicionadas por la enfermedad inmunosupresora con pronóstico reservado. El objetivo de este reporte es describir un caso de equinococosis multiquística peritoneal en una paciente en tratamiento antiviral para VIH durante casi diez años, que recibió la combinación de albendazol más cirugía, con evolución favorable. Este reporte sería el primero en el Perú en una persona con inmunosupresión por VIH y equinococosis quística.

## INTRODUCCIÓN

La equinococosis quística (EQ) es una infección zoonótica de impacto mundial producida por la forma larvaria de la tenia *Echinococcus granulosus* que se encuentra en los huevos del parásito, los que contaminan suelo, agua y alimentos a través de las heces que elimina el perro, quien es el hospedero definitivo ^(^^1^^,^^2^. El hospedero intermediario (algún tipo de ganado) y el ser humano se infectan a partir de los elementos contaminados. Cuando la larva llega al intestino se libera para atravesar la pared intestinal e invadir diversos órganos, preferentemente el hígado y los pulmones ^3^.

Esta enfermedad es propia de áreas donde se cría principalmente ganado ovino y bovino, y tiene una alta incidencia en la región mediterránea, norte de África, sur y este de Europa, Sudamérica, Asia central, Siberia y occidente de China ^4^. En las regiones endémicas, la incidencia puede alcanzar los 50 casos por cada 100 000 habitantes por año, y su prevalencia en áreas específicas del Perú y otros países de Sudamérica puede variar entre 5 y 10% ^5^. Aproximadamente el 60% de los casos en Perú provienen de los departamentos de Lima, Huancavelica, Junín, Cerro de Pasco y Cusco, ocurren principalmente en mujeres adultas, y un tercio tienen localización pulmonar ^6^.

La coinfección con parásitos suele ser una complicación grave para los pacientes infectados por el virus de la inmunodeficiencia humana (VIH), sin embargo, son pocos los reportes de coinfección con EQ a nivel mundial ^7^. Por ello, describimos el primer caso en Perú, destacando el manejo interdisciplinario de una enfermedad con evolución acelerada y múltiples complicaciones en los pacientes inmunosuprimidos.

## REPORTE DE CASO

Mujer de 34 años, natural del distrito de Paucartambo, provincia de Pasco, región de Pasco en Perú, con antecedente de infección por VIH en tratamiento irregular con dolutegravir/lamivudina/tenofovir desde el 2013, refiere tener un hijo tratado quirúrgicamente el 2022 por hidatidosis hepática abscesificada y esplénica. Actualmente la paciente reside en Lima, pero manifiesta contacto con ganado bovino y perros en su comunidad de origen. Desde el 2013, la paciente refiere un cuadro clínico caracterizado por dolor abdominal leve, con resultado de ultrasonografía donde se reporta una masa multiquística abdomino-pélvica de aproximadamente 10 cm. Desde el 2020, la paciente reporta dolor abdominal y un aumento de diámetro abdominal, náuseas, vómitos y constipación. Durante los últimos meses del 2021, se agregó edema de miembros inferiores y dificultad para tolerar la vía oral, por lo que se realiza una prueba de Western Blot, que resulta para *Echinococcus granulosus*, por lo que se inició el primer ciclo de tratamiento con albendazol 400 mg cada 12 horas por 28 días. En la Figura 1 se muestra la evolución progresiva del tamaño y complejidad de las masas quísticas mediante los estudios de imagen.

**Figura 1:** Estudios de imagen. (A) Ultrasonografía realizada en el 2022: masa heterogénea, de 9 × 10 cm2, con múltiples imágenes quísticas en su interior, de clasificación Gharbi III. (B) Corte sagital de tomografía espiral multicorte con contraste. (C) corte coronal de tomografía espiral multicorte con contraste rea-lizada en el 2022: masa de aspecto quístico complejo de naturaleza mixta de 25 × 19 × 26 cm3, con desplazamiento de estructuras intraabdominales adyacentes e hidroureteronefrosis, de clasificación Gharbi tipo IV.

A inicios del 2022, la paciente fue evaluada y admitida en el servicio de cirugía, iniciándose un segundo ciclo con albendazol. Adicionalmente, se le indicó suplementación oral preoperatoria con fórmula hipercalórica e hiperproteica más inmunonutrientes y hierro polimaltosado por cuatro semanas, debido a un cuadro de desnutrición y anemia. Posteriormente, la paciente fue sometida a una laparotomía exploratoria de emergencia, debido a una obstrucción intestinal, realizándose la resección quirúrgica completa de una masa multiquística compleja de 35 × 30 × 30 cm^3^, de paredes engrosadas y fusionadas con el omento mayor, que se extendía hacia pelvis y retroperitoneo, adherida firmemente al peritoneo anterior y posterior, cara posteroinferior de la vejiga y polo superior de útero (Figura 2). Adicionalmente, se resecaron múltiples lesiones quísticas menores de 8 cm, localizadas en toda la raíz mesentérica del yeyuno, íleon y colon, y se colocaron dos drenajes tubulares siliconados, en recesos laterales intraabdominales.

**Figura 2:** Masa quística compleja. (A) Pieza operatoria y membranas vesiculares (punta de flecha). (B) Distensión abdominal provocada por ocupación de la masa quística intraabdominal. (C) Lesiones quísticas múltiples (flechas) que ocupan el mesenterio intestinal.BCABC

La estancia hospitalaria fue de siete días, donde la paciente recibió terapia antibiótica (ceftriaxona + metronidazol), tercer ciclo con albendazol, transfusión de cinco paquetes globulares por anemia aguda poscirugía, evolucionando favorablemente con adecuada tolerancia oral, además de suplementación calórico-proteica, drenajes tubulares con débito serohemático, hasta su retiro en el control, luego del alta hospitalaria.

La histopatología de los especímenes reportó un quiste hidatídico complejo viable (Figura 3). En la Figura 4 se muestra la evolución clínica y los resultados de laboratorio. Luego de seis meses de la cirugía, la paciente realiza sus actividades normalmente, cumple con su tratamiento antirretroviral y acude a sus controles periódicos por consultorio externo.

**Figura 3:** Histopatologia de la pieza operatoria. Secciones histologicas del quiste, tincion hematoxilina-eosina. (A) La pared del quiste tiene tres componentes estructurales: capsula fibrosa (f), membrana laminada acelular (m) y membrana germinativa (g) (10X). (B) Protoescolices (p) que brotan de la membrana germinativa (4X). (C) Protoescolices con ventosas y ganchos (flecha) (40X).

**Figura 4:** Comparación de resultados de laboratorio pre y poscirugía.

## DISCUSIÓN

La EQ es una enfermedad endémica en muchas zonas del mundo, relacionada con las actividades ganaderas de la población que, sumadas al limitado conocimiento del mecanismo de trasmisión y a las deficientes medidas de prevención, condicionan la persistencia de la zoonosis ^(^^4^. El desarrollo asintomático y lento de la enfermedad, puede desencadenar en el crecimiento abrupto de las lesiones quísticas, y el compromiso de múltiples órganos en un 20% ^3^.

Respecto a la sintomatología, lo más frecuente es el dolor abdominal ^8^, casi siempre, asociado a las complicaciones propias del tamaño del quiste. En nuestro caso, las dimensiones abruptas de la masa multiquística ocasionaron la compresión de múltiples órganos (estómago, asas intestinales, uréteres y vejiga), y la aparición de síntomas como anorexia, náuseas y vómitos, de forma similar a otros reportes descritos en la literatura ^8^^-^^12^, además de la presencia de incontinencia de esfuerzo y edemas de miembros inferiores, como síntomas poco comunes.

Recientemente, se ha informado que el VIH es la infección inmunodeficiente más frecuente en pacientes con EQ ^2^. Además, algunos reportes indican que la enfermedad quística extrahepática es más común en la población con VIH ^7^, tal como se ha descrito en casos con localizaciones poco comunes como cerebro ^13^, médula espinal ^14^, globo ocular ^15^, renal ^16^, hepatorrenal ^17^ y peritoneal ^18^. La mayor parte de estos casos han ocurrido en mujeres. ^2^.

Los mecanismos de inmunidad implicados en la defensa frente a la equinococosis se caracterizan por la activación de las células Th2 y un subconjunto de Th1, lo que permite la expresión de isotipos de inmunoglobulina (IgG4 e IgE) y eosinofilia ^19^. En nuestro caso, se pudo notar una eosinofilia transitoria posterior a la intervención quirúrgica, lo que se podría explicar por la ruptura de algunos quistes durante la extirpación quirúrgica ^12^, considerando que el estudio de anatomía patologíca reportó presencia de quistes viables.

En el contexto del tratamiento de la EQ con albendazol, diferentes estudios reportan una mejoría de los síntomas y una disminución radiológica del tamaño del quiste e incluso seronegatividad ^1^, esto podría deberse al efecto del tratamiento antiparasitario en el aumento de la producción de interferón gamma (IFN-γ) y factor de necrosis tumoral alfa (TNF-α) por células mononucleares periféricas ^19^. Contrariamente, en nuestro caso se pudo evidenciar un aumento abrupto del tamaño de la masa quística con progresión tórpida de la evolución clínica, debido a la compresión gástrica, intestinal y ureteral, concordante con una disminución de células T CD4+ (55 cel/uL) durante el tratamiento con albendazol. Estos hallazgos se explican debido a la supresión de estas células que ocasiona la infección parasitaria ^20^, lo que predispone la proliferación no controlada de protoescólices, crecimiento acelerado y la formación de multiquistes en pacientes VIH positivos con inmunidad alterada ^12^, y en este caso, en un estadio de síndrome de inmunodeficiencia adquirida (SIDA).

Una revisión sistemática reportó que la cirugía es el tratamiento principal para la EQ en pacientes inmunocomprometidos, siendo realizado en más del 70% de los casos ^2^. En nuestro caso, se optó por un tratamiento quirúrgico en coadyuvancia con manejo antiparasitario, debido a que la evidencia indica que la combinación de ambas intervenciones puede incrementar significativamente el recuento de células T CD4+ ^8^^,^^20^, disminuyendo la morbilidad asociada. Sin embargo, cuando las lesiones alcanzan un tamaño considerable, la mortalidad se asocia principalmente a complicaciones propias de las intervenciones ^3^, en nuestro caso, la anemia aguda por pérdidas durante la cirugía fue controlada con transfusiones.

La principal limitación del presente caso fue no haber realizado una actualización del recuento de células T CD4+, debido a que este examen se realiza en el Instituto Nacional de Salud, mediante referencia y previa evaluación en el consultorio de infectología del Hospital Hipólito Unanue, situación que toma demasiado tiempo. Adicionalmente, debido a la complejidad de la lesión, la paciente fue diferida a distintas especialidades e instituciones de salud, situación que prolongo la intervención quirúrgica. Posiblemente, ambos factores hubieran permitido un mejor manejo de la paciente.

Luego de ocho meses de seguimiento, la paciente no reportó ninguna intercurrencia luego de la intervención quirúrgica. Por lo mencionado, se concluye que una terapia combinada (cirugía-antiparasitario) seria de elección frente al manejo médico, en el tratamiento de la EQ en pacientes coinfectados con VIH/SIDA, con resultados favorables a largo plazo.
